# Midshaft clavicle fractures with associated ipsilateral acromioclavicular joint injuries: a systematic review

**DOI:** 10.1186/s12893-025-02815-x

**Published:** 2025-02-28

**Authors:** Chaoqun Wang, Xugui Li, Shengnan Dong, Wei Xie, Zexi Ling, Chengfei Meng, Ulrich Stöckle

**Affiliations:** 1 Department of Traumatic Orthopaedics, The Affiliated Hospital of Wuhan Sports University, Hongshan District, NO.279 On Luoyu Road, Wuhan CityHubei Province, 430079 China; 2 Center for Musculoskeletal Surgery, Charité – Universitätsmedizin Berlin, corporate member of Freie Universität Berlin and Humboldt-Universität Zu Berlin, Augustenburgerplatz 1, 13353 Berlin, Germany

**Keywords:** Clavicle fracture, Acromioclavicular joint, Midshaft clavicle, Bipolar clavicle injuries, Coracoclavicular ligament

## Abstract

**Background and aim:**

Isolated midshaft clavicle fractures (MCF) and acromioclavicular joint (ACJ) injuries are common, but simultaneous cases are rare and often receive insufficient clinical attention, resulting in missed diagnoses. Moreover, there is no consensus on the injury mechanism, classification, and treatment, and the prognosis remains poorly summarized. This review aims to provide an overview of MCFs with ipsilateral ACJ injuries, focusing on injury mechanism, classification, treatment, and prognosis.

**Methods:**

We searched the literature published between 1962 and 2024 on PubMed, Web of Science, and EMBASE using the search terms “clavicle fracture [Title/Abstract]) AND (acromioclavicular [Title/Abstract])”. Studies reporting clinical outcomes in patients with MCF and ipsilateral ACJ injuries were included. 37 studies were included after screening. The study quality was assessed using the Joanna Briggs Institute Critical Appraisal Checklist. Data on study design, patient demographics, treatment approaches, and outcomes were extracted for qualitative analysis. We then summarized key findings and presented our insights.

**Results:**

MCFs with ipsilateral ACJ injuries are often associated with comorbidities such as rib fractures, hemopneumothorax, scapula fractures, neurovascular injuries, and atypical MCF displacement patterns. These cases should raise suspicion for combined injuries. Due to the "floating" nature of the lateral clavicle, the "Piano Key Sign" is typically negative and not reliable for diagnosis. Initial ACJ evaluation may be inconclusive, so reevaluation after MCF fixation is recommended. Type IV ACJ injuries can be underestimated on anteroposterior radiographs, and additional axillary radiographs and CT scans may better visualize posterior clavicle displacement. Most researchers believe ACJ capsule and ligament damage occurs first, but is insufficient to cause significant dislocation, suggesting that isolated MCF may involve combined ACJ injury with intact coracoclavicular ligaments. Notably, most patients reported favorable outcomes without major complications within two years, regardless of treatment approach.

**Conclusions:**

MCFs with ipsilateral ACJ injuries are rare and often missed when ACJ injuries are mild. The injury mechanism is unclear, and no classification system exists to indicate severity. These injuries are typically treated separately without a unified protocol. Despite promising outcomes, further studies are needed to address these issues and improve understanding of long-term results.

## Introduction

Midshaft clavicle fractures (MCF) and acromioclavicular joint (ACJ) injuries are common when occurring separately, but their simultaneous occurrence is rare. Due to this rarity, clinical attention is often insufficient, leading to a high risk of missed or misdiagnoses, especially when ACJ injuries are mild. This can result in progressive dislocation, arthritis, and pain, often requiring revision treatments [1, 2]. Additionally, while the principles and modalities of treatment for both MCF and ACJ injuries are well established [3–9], a unified approach for simultaneous injuries remains unclear and may not be as simple as a "1 + 1 = 2" solution. Also, the prognosis following different treatments had not been well summarized in the past few years. To improve the understanding of MCF with concomitant ipsilateral ACJ injuries, which is crucial for enhancing patient outcomes, we reviewed relevant literature, summarized key findings, and provided an overview along with our insights into this rare injury. This review focuses on the injury mechanism, classification, treatment, and prognosis, aiming to raise clinical awareness, reduce underdiagnosis, and ultimately benefit patients.

## Methods

We conducted a literature search with reference to the PRISMA 2020 statement [10]. Ethical approval was not required for this review of publicly available data.

## Database and searching strategies

We performed a comprehensive literature search in the electronic databases of PubMed, Web of Science, and EMBASE with no language restrictions. The publication dates were limited from January, 1962 to June, 2024. Search terms in different databases are shown in Table 1. After the electronic search was completed, the relevant literature and references were searched manually to find potential eligible studies.

**Table 1:** Search strategies for PubMed, Web of Science, and EMBASE

Databases	Searching terms	Time limits
PubMed	(clavicle fracture[Title/Abstract]) AND (acromioclavicular[Title/Abstract])	1962–01-01 TO 2024–06–15
Web of Science	((TI = (clavicle fracture)) OR (AB = (clavicle fracture))) AND ((TI = (acromioclavicular)) OR (AB = (acromioclavicular)))	1962–01-01 TO 2024–06–15
EMBASE	'clavicle fracture':ab,ti AND acromioclavicular:ab,ti AND [< 1966–2024]/py	1962–01-01 TO 2024–06–15

## Inclusion criteria

We follow the population/intervention/comparator/outcome/study design (PICOS) principle to develop the inclusion criteria [11]. (1) Population: patients were diagnosed with MCF combined ipsilateral ACJ injury. (2) Intervention: patients were treated conservatively or surgically. (3) Comparator: not essential. (4) Outcomes: studies had at least one of the following clinical outcomes, including functional outcome, complications, reduction of joint luxation, and bony union. (5) Study design: no limitation.

## Exclusion criteria

Our exclusion criteria are as follows: (1) Studies that do not provide concrete patient and treatment-related information, such as review articles, systematic reviews, surgical techniques, guidelines, textbooks, and cadaveric studies; (2) Studies where the patients' primary diagnoses differ from the topic of our study.

## Screening, study selection and data extraction

The search results were imported into EndNote 19.0 (Clarivate Analytics, Philadelphia, PA, USA), and duplicates were removed. Subsequently, three authors (CW, SD, CM) independently reviewed the titles and abstracts of each article. In case of disagreements, a fourth independent author (ZL) assessed the article. The articles that potentially met the inclusion criteria were further analyzed, and only those meeting all inclusion criteria were used for analysis. The following data were extracted from all included studies: study details (study design, authors, publication year, country), patient demographics (age, gender, affected side), and management characteristics (surgical or conservative, immobilization strategies, implants, and rehabilitation), clinical outcomes (follow-up time and results, functional outcome, postoperative complications, ACJ alignment, and bony union).

## Quality assessment

As most of the included were case reports, two authors (XL and WX) assessed their quality using the Joanna Briggs Institute (JBI) Critical Appraisal Checklist for Case Reports [12], which comprises eight questions designed for objective quality assessment. Evaluators are required to respond to each question with "Yes," "No," "Unclear," or "Not Applicable" based on the actual content of each study. While the original JBI checklist does not employ a conventional scoring system, we established a quantitative evaluation method by assigning 1 point for each "Yes" response and 0 points for other responses. Based on this scoring system, studies were categorized as follows: excellent (6–8 points), good (3–5 points), and poor (0–2 points). Any discrepancy was solved by a discussion with third independent author (CW).

## Data analysis

Given the nature of the included studies, which primarily consist of case reports, statistical analysis was not feasible. The small sample sizes, heterogeneous patient populations, and variations in treatment approaches across studies made it difficult to perform meaningful quantitative analyses. As such, a qualitative approach was adopted for this study. The focus of this review was to synthesize and summarize the findings from the case reports, rather than to conduct statistical comparisons. A qualitative analysis allows for a comprehensive understanding of the clinical outcomes, treatment strategies, and complications reported in individual cases, which are essential for drawing insights from case-based literature. We have provided a narrative synthesis of the data, including detailed descriptions of patient demographics, treatment modalities, and outcomes, in order to highlight trends and commonalities across studies. Thus, the decision to use a qualitative approach was based on the limitations of the available data, and it was deemed the most appropriate method to provide a meaningful synthesis of the clinical findings in this context.

## Results

**Fig. 1:**
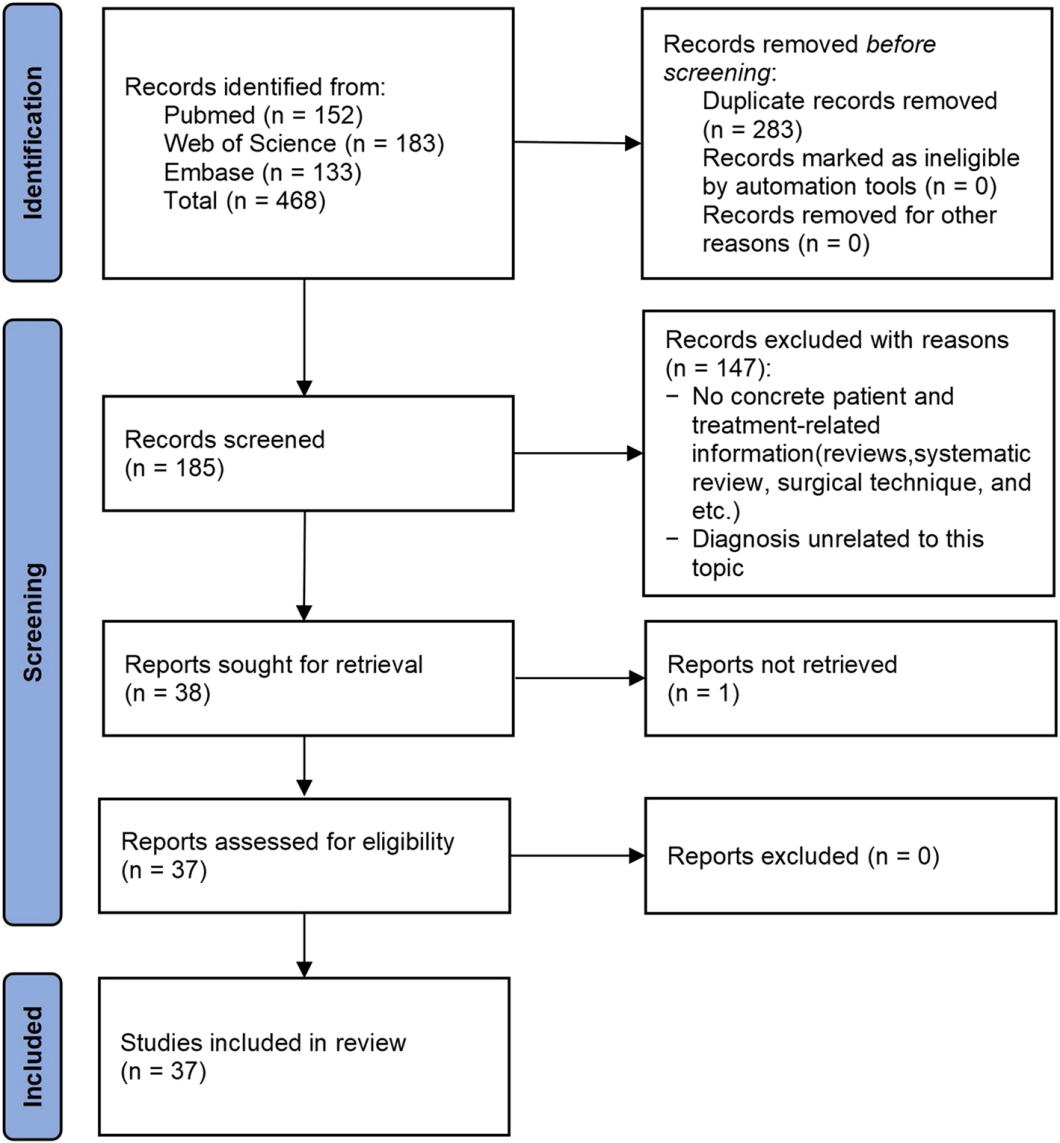
PRISMA diagram showing the screening process

After careful screening, we identified a total of 38 studies related to MCF with associated ipsilateral ACJ injuries between 1962 and 2024 [1, 2, 13–48]. It should be noted that three case reports were unavailable in full text, but two [46, 47] of them provided detailed case descriptions in the abstracts, so they were included. However, another study from Japan [37] was excluded. Therefore, this review is primarily based on the remaining 37 studies. The specific inclusion process flowchart is shown in Fig. 1.

Almost all of the included studies were published in English by authors from 18 countries, with the exception of Sebesta et al. [36] who published a case report in Czech. The largest number of studies came from the United States, with 11 studies reporting a total of 40 cases. Although only one study was from Germany, it reported the highest number of cases, with a total of 106 cases [26]. In terms of chronological order, the first study on MCF with associated ipsilateral ACJ injuries was published in the year of 1990 by Lancourt [43], who reported a horseback rider suffered this rare injury. Since 2010, the number of recorded MCF with associated ipsilateral ACJ injuries has increased considerably, whereas prior to 2010 there were only 6 studies, most likely due to the increased speed of vehicles making people more vulnerable to high-energy trauma as well as an increased awareness of such combined injuries.

34 [1, 2, 13, 14, 16–25, 27–31, 33–36, 38–48] out of 37 studies were case reports with a detailed description including radiologic records, treatment modalities, rehabilitation therapies, and follow-up results, which allowed for a critical analysis of each case. Three exceptions, which mainly focused on the incidence of bipolar clavicle injuries, were published by Bakir et al. [26], Ottomeyer et al. [15], and Chillemi et al. [32], respectively. The key findings and quality assessment results for each case report are presented in Table 2.

**Table 2:** Summary of previous 34 case reports

**Authors, Year**	**N**	**Gender, age,** **Side (M/F, y,** **R/L)**	**Injury mechanism**	**Associated injury**	**ACJ dislocation**		**MCF treatment**	**Follow-up**	**Complications**	**Results**	**Additional information**	Study quality^**^
					**Type**	**Treatment**						
López Palacios et al. [13], 2021	1	M, 41, L	Fall from a bicycle	None	IV	2 suture anchors inserted into coracoid process	Locking plate	2.5 years	Transient hypesthesia in surgical scar for 1 month after surgery	Asymptomatic Full ROM Constant-Murley score 97	No hardware removal	8
Prasetia et al. [14], 2017	1	M, 32, R	Traffic accident	Multiple rib fractures Ipsilateral coracoid process fracture and SCJ dislocation	V	Semitendinosus tendon grafting Kirschner wires	One compression screw Locking plate	0.5 years	None	Asymptomatic Full ROM ASES score 84	Kirschner wire removed 3 weeks after surgery Surgery done 23 days after injury	8
Kembhavi, et al. [16], 2015	1	M, 38, R	Traffic accident	Ipsilateral scapular spine and acromion fracture	NS	Conservative	Locking plate	7 months	Mild ACJ separation	Asymptomatic Full ROM Constant-Murley score 97	None	8
Gao et al. [17],2021	1	M, 65, R	Traffic accident	Hemopneumothorax Multiple rib fractures	V	Endobutton Kirschner wires	Locking plate	6 months	None	Asymptomatic Constant-Murley score rates 92	None	8
Fulton et al. [1], 2022	1	M, 37, L	Fall from a bicycle	None	III	Knotless TightRope	Locking plate	6 months	None	Asymptomatic Full ROM	Ipsilateral ACJ dislocation found 6 weeks after clavicle fixation	8
Cosic et al. [18], 2022	1	M, 30, L	Fall from a bicycle	None	V	Hook plate Suture anchor	Lag screw Locking plate	6 months	None	Asymptomatic Full ROM ASES score 90	Hook plate removed 4 months after surgery	8
Marjoram et al. [19],	1	M, 40, L	Fall from a motorcycle	None	V*	Hook plate	Locking plate	8 months	None	Asymptomatic Full ROM Oxford Shoulder Score 45/48	Clavicle fracture diagnosed during surgery Hook plate removed 4 months after surgery	7
Shih et al. [20], 2023	1	M, 60, L	Traffic accident	None	V	Knowles pin	Knowles pin	1 year	None	Asymptomatic Full ROM Constant-Murley score 96	ACJ dislocation diagnosed during surgery Implants removed 4 months after surgery	8
Lee et al. [21], 2018	1	M, 50, R	Fall from a 4-m ladder	Multiple rib fractures Hemopneumothorax	III	Conservative	Conservative	10 months	Mild ACJ widening with slight pain Mild clavicle deformity	Constant-Murley score 83	None	8
Solooki et al. [22], 2014	1	M, 40, L	Traffic accident	None	III	2 CC screws	Locking plate	1 year	None	Asymptomatic Full ROM	None	8
van de Voort et al. [23], 2022	1	F, 55, R	Traffic accident	None	IV	TightRope Suture anchors	Locking plate	6 months	None	DASH score 0 Full ROM	None	8
Grossi et al. [24], 2013	1	M, 19, R	Fall from a bicycle	None	VI	Steinmann’s pins	Conservative	12 months	None	Excellent clinical and radiological results	Implants removed 7 weeks after surgery Intact CCL	8
Schots et al. [25], 2020	2	M, 43, R	Fall from a 2-m scaffold	None	IV	Hook plate Suture anchors Mesh	Locking plate	5 months	ACJ osteoarthritis with ongoing pain	Full ROM	The plates removed 5 months after surgery Further lateral clavicle resection was performed	8
		M, 34, R	Fall from a bicycle	None	III	Hook plate Suture anchors Mesh	Locking plate	7 months	None	Asymptomatic Full ROM	Hook plate removed 7 months after surgery	8
Sharma et al. [27], 2016	1	M, 65, L	Traffic accident	Ipisilateral humerus and coracoid fracture	III	Kirschner wires and stainless steel wire in a figure-eight manner	Locking plate	6 months	None	Asymptomatic Full ROM Constant-Murley score 92	Implants removed 6 weeks after surgery	8
Psarakis et al. [28],	1	M, 38, R	Traffic accident	None	V	TightRope	Locking plate	18 months	None	Asymptomatic Full ROM	None	8
Park et al. [29], 2016	1	M, 55, L	Fall from 7-feet height	Multiple rib fractures	III	Conservative	Locking plate	15 months	ACJ osteoarthritis	Full ROM	None	8
Yeh et al. [30], 2019	1	F, 46, R	Fall from a horse	None	IV*	Semitendinosus tendon grafting	Locking plate	1 year	None	Asymptomatic Full ROM	None	7
Davies et al. [31], 2014	1	F, 40, R	Fall from stairs	Intermittent paraesthesia of the right hand Occasional transient blue or white discoloration of the right arm	VI	Conservative	Lag screw Locking plate Iliac crest autograft	9 months	None	Asymptomatic Full ROM	ACJ was spontaneously reduced after clavicle fixation	8
Madi et al. [33], 2015	1	M, 21, L	Traffic accident	Right humerus fracture Multiple thoracic transverse process fractures	IV	Arthroscopy Dog bone button Fibre tape	Locking plate	13 months	None	Asymptomatic Full ROM Constant-Murley score 88	None	8
Mohammed et al. [2], 2016	1	M, 33, L	Fall from a bicycle	None	IV	Synthetic CC sling augmented by a cortical screw	Locking plate Local bone graft	22 months	None	Asymptomatic Full ROM ASES score 100 Constant-Murley 93	ACJ injury missed initially Surgery 25 months after injury	8
Juhn et al. [34], 2002	1	M, 21, R	Struck the boards with shoulder	Transient tingling sensation in the right arm	VI	Conservative	Conservative	10 months	Slight clavicle angulation and distal clavicle osteolysis	Asymptomatic Full ROM	None	8
Dong et al. [35], 2016	1	F, 42, L	Traffic accident	Hemopneumothorax Right clavicle fracture	IV*	Hook plate	Locking plate	1 year	None	Asymptomatic Full ROM	Bilateral midshaft clavicle fractures	7
Sebesta et al. [36], 2014	1	F, 46, R	Fall from a bicycle	None	IV*	Kirschner wires and stainless steel wire in a figure-eight manner	Locking plate	1 year	Morning stiffness in the right shoulder	Full ROM	None	7
Beytemür et al. [38], 2013	1	M, 50, L	Traffic accident	First and second costa fracture	III	Hook plate	Low profile anatomic clavicle plate	23 months	ACJ widening and degeneration	Asymptomatic Full ROM	The hook plate was not removed	8
Heinz et al. [39], 1995	1	M, 34, L	Fall from a bicycle	None	III*	Conservative	Conservative	24 months	Deformity of the mid and distal clavicle Wide ACJ separation	ROM equal to the unaffected side	None	7
Wijdicks et al. [40], 2013	2	M, 44, R	Fall from a motocross bike	Multiple rib fractures Pneumothorax	III	Hook plate	Lag screws Superior locking plate Anterior reconstruction plate	13 months	Mild ACJ separation	Full ROM DASH score 3.33	Hook plate removed 4 months after surgery	8
		M, 36, L	All-Terrain Vehicle rollover	Right-sided acetabular fracture and scapulothoracic dissociation	IV	Hook plate	Locking plate	6 months	None	Asymptomatic Nearly normal ROM DASH score 30	Hook plate removed 6 months after surgery	8
Wisniewski [41], 2004	1	M, 32, L	Struck by a car	None	IV*	Kirschner wires	Conservative	10 years	None	Asymptomatic Full ROM	Stretched but intact CCL	7
Sobhani Eragh et al. [42], 2020	1	M, 29, L	Fall from a bicycle	None	IV	Endobutton	Anatomical clavicle plate	1 year	Slight loss of ACJ reduction	Asymptomatic Full ROM	Started unauthorized sports activities too early	8
Lancourt [43], 1990	1	F, 19, L	Fall from a horse	None	V*	Steinmann’s pins	Conservative	3 years	None	Asymptomatic Full ROM	Steinmann’s pins removed 8 weeks after surgery	7
Milchteim et al. [44], 2018	1	M, 18, L	Fall from a motorcycle	None	VI*	Knowles pin	Knowles pin	12 weeks	None	Asymptomatic Full ROM ASES score 95	Fracture was spontaneously reduced after reducing ACJ	7
Kakwani et al. [45], 2011	1	M, 45, L	Traffic accident	None	IV*	Tightrope	Locking plate	5 months	None	DASH score 11.7 Full ROM	None	7
Woolf et al. [46], 2013	1	NA	Traffic accident	NA	IV	ORIF Implants NA	ORIF Implants NA	NA	NA	Satisfactory patient-reported outcomes	Abstract available only	4
Tidwell et al. [47], 2014	1	M, 19, NA	NA	NA	IV	CC screw	Locking plate	1 year	NA	Returned to manual labor and normal activities of daily living	Abstract available only CC screw was removed 3 months after surgery	5
Wurtz et al. [48], 1992	4	M, 36, L	Fall from a bicycle	NR	IV	CC screw	Conservative	3 years	None	Asymptomatic Full ROM	CC screw was removed 8 weeks after surgery	7
		M, 23, R	Traffic accident	NR	IV	CC screw	Osteoclasis Implants NA	2 years	None	Asymptomatic Full ROM	None	6
		F, 19, NR	Fall from a horse	NR	IV	Steinmann’s pins traversing ACJ	Conservative	3 years	None	Asymptomatic Full ROM	None	6
		F, 33, NR	Fall from a horse	NR	II	Conservative	Conservative	1 year	None	Asymptomatic Full ROM	None	6

*M* male, *F* female, *R* right, *L* left, *ACJ* acromioclavicular joint, *MCF* midshaft clavicle fracture, *ROM* range of motion, *SCJ* sternoclavicular joint, *ASES* American Shoulder and Elbow Surgeon, *CC* coracoclavicular, *DASH* Disabilities of the arm, shoulder, and hand, *CCL* coracoclavicular ligament, *NS* not suitable, *NA* not available, *NR* not recorded, *ORIF* open reduction and internal fixation

^*^The ACJ dislocation classification was inferred from radiological findings, as the original text lacked explicit description

^**^Study quality was assessed using the Joanna Briggs Institute Critical Appraisal Checklist for Case Reports, with a maximum score of 8 points: excellent (6–8 points), good (3–5 points), and poor (0–2 points)

## Discussion

Through a systematic synthesis of the primary findings from the included studies and based on clinical experience, our study comprehensively aims to discuss the clinical issues related to MCFs with ipsilateral ACJ injuries and offer some insights to advance the understanding of this uncommon injury.

## Pathoanatomy and mechanism of injury

**Fig. 2:**
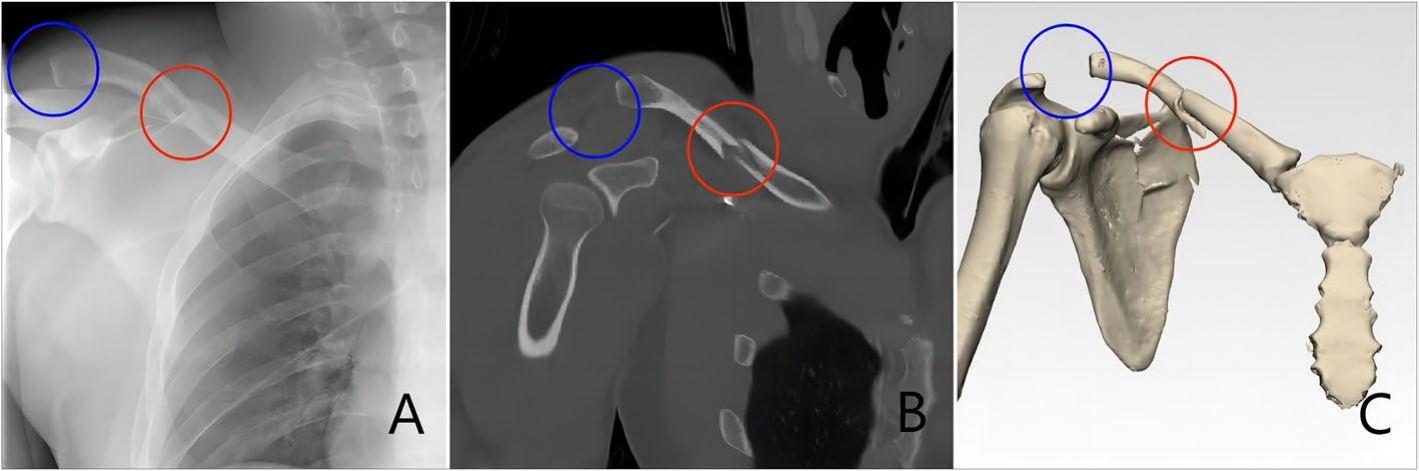
Typical radiologic findings in midshaft fractures with associated ipsilateral acromioclavicular joint injuries. (**A**) AP view. (**B**) Coronal section of CT scan; (**C**) 3D CT reconstruction. Blue circle, type V acromioclavicular joint dislocation; red circle, displaced midshaft clavicle fracture

MCFs with associated ipsilateral ACJ injuries represent a complex injury pattern involving multiple structures surrounding the clavicle and ACJ. Radiologic findings typically show marked clavicular displacement and ACJ malalignment in this condition(Fig. 2). The clavicle, as the only bony connection between the trunk and upper limb, plays a crucial role in shoulder mechanics. Its palpable, gentle S-shaped contour consists of a forward-facing convex medial portion and a concave lateral portion. MCFs typically occur at anatomical transition zones, most commonly caused by falls onto an outstretched hand or direct impact [6–9, 49, 50]. In contrast, the ACJ, surrounded by a joint capsule and reinforced by the acromioclavicular ligament (ACL) and the stronger coracoclavicular ligament (CCL), is less frequently injured but often results from similar mechanisms of trauma [51, 52]. Furthermore, the high-energy nature of such trauma often causes associated injuries, including rib fractures, hemopneumothorax, scapular fractures, and neurovascular injuries. The presence of these associated injuries should therefore raise clinical suspicion for concurrent ipsilateral ACJ dislocation in patients with clavicle fractures.

Importantly, based on the reviewed cases, radiographic findings in combined MCFs with ipsilateral ACJ injuries frequently deviated from typical fracture displacement patterns observed in isolated MCFs [14, 18, 23–25, 28, 30, 34, 40, 44, 45, 48]. Additionally, the concurrent involvement of both structures complicates radiographic assessment, as the actual severity of ACJ frequently demonstrate inconsistencies with the corresponding classification types in the Rockwood system [1, 16, 20, 24, 29, 41]. These findings highlight the necessity for further investigation into the unique biomechanical mechanisms underlying such combined injuries.

However, due to the rarity of these combined injuries in clinical practice, a consensus regarding their precise mechanism remains elusive, primarily due to the lack of high-level evidence. Available case reports indicate that high-energy trauma, particularly falls from two-wheeled motorcycles, is a predominant cause. This suggests that a combination of direct shoulder blows and simultaneous rotation may play a critical role in the pathogenesis of these complex injuries.

Two main hypotheses exist for the mechanism behind bipolar clavicle injuries [21]. One proposes that both injuries occur simultaneously, while the other suggests a consecutive occurrence. Most experts believe that MCFs with associated ACJ injuries typically occur consecutively, though the exact sequence remains controversial [19, 23, 33, 41].

Marjoram et al. [19] and van de Voort et al. [23] suggested that high-energy trauma firstly causes ruptures of both the ACL and CCL, leading to ACJ dislocation. Subsequently, the force is transmitted medially, resulting in clavicle fracture. Similarly, Okano et al. [53] introduced the "first rib pivot theory," which postulates that a posterior force applied to the ACJ results in dislocation, with the first rib acting as a pivot point for the subsequent clavicle fracture or sternoclavicular joint (SCJ) dislocation. All these theories imply that ligament ruptures occur before the clavicle fractures. However, documented cases demonstrating ACLs rupture with intact CCLs have been reported, challenging these hypotheses [16, 24, 41].

Wisniewski [41], on the other hand, proposed an alternative mechanism: a shoulder collision causing the scapula to displace forward and upward relative to the clavicle. This first damages the ACJ capsule, ACL, and surrounding muscle fascia, and then, if the energy is sufficient, transmits medially to cause a clavicle fracture. In this scenario, the CCL remains intact until the clavicle fractures. This hypothesis is supported by cases where the CCL was found intact during surgery, and ACJ stability was restored following clavicle fixation [16, 24, 31, 41, 44]. Other studies, including those by Fulton et al. [1], Park et al. [29], and Wurtz et al. [48], support this idea that when the CCL remains intact, a downward force on the shoulder can cause a clavicle fracture, followed by ACJ dislocation if sufficient residual force is applied. Nevertheless, While Wisniewski's hypothesis provides a more comprehensive explanation for MCFs with associated ipsilateral ACJ injuries compared to the theories proposed by Marjoram et al. [19], van de Voort et al. [23], and Okano et al. [53], it fails to account for isolated ACJ dislocations that occur without associated clavicle fractures despite complete rupture of both the ACLs and CCLs.

**Fig. 3:**
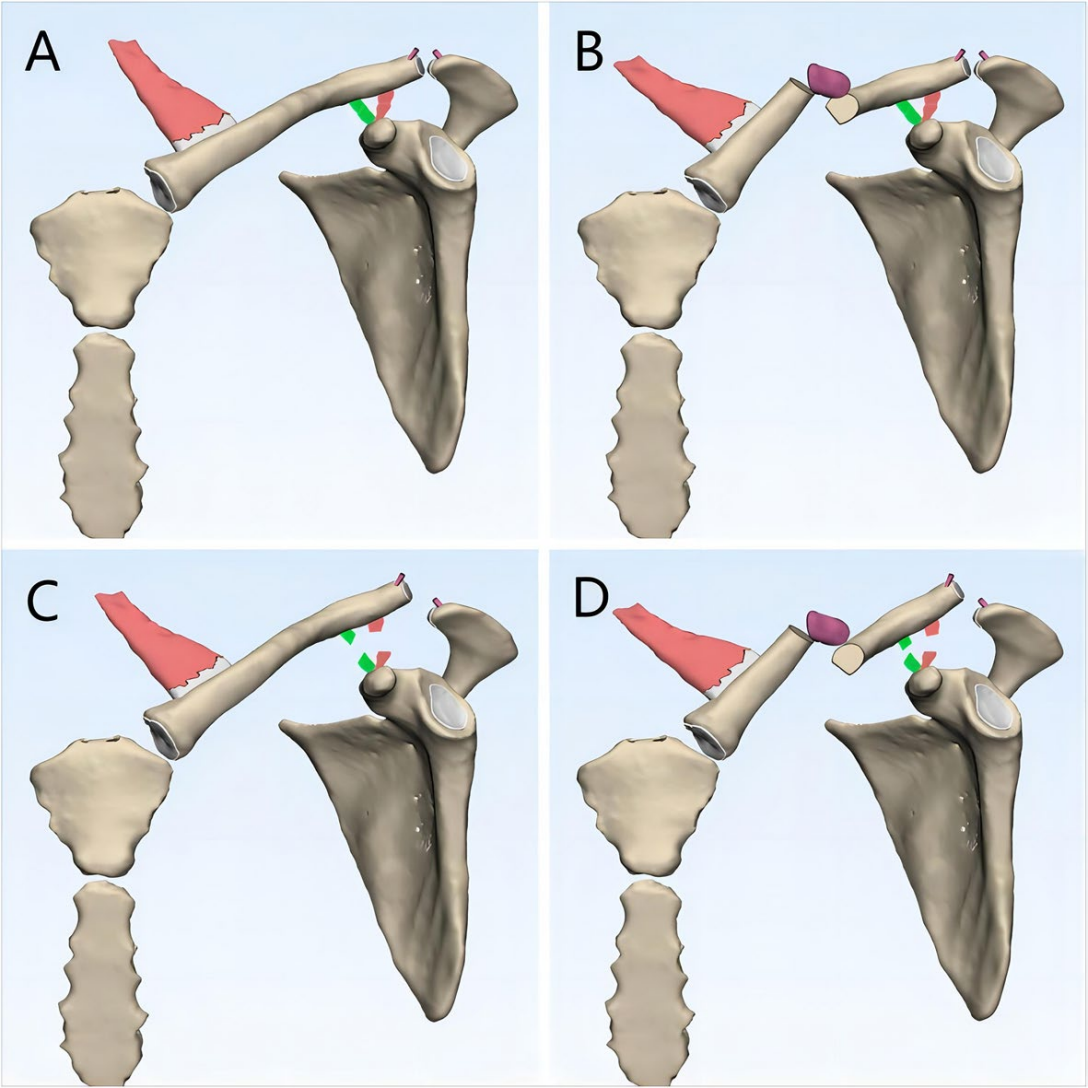
Diagram of the proposed mechanism of injury. (**A**) Injury to the acromioclavicular ligament and articular capsule is the first step in the development of the injury; (**B**) if the coracoclavicular ligament is more resistant to traumatic energy than the clavicle, the clavicle fracture will occur at the second step, resulting in an isolated clavicle fracture if the acromioclavicular joint dislocation does not occur in the next step; (**C**) conversely, if the clavicle is more resistant to traumatic energy than the coracoclavicular ligament, rupture of the coracoclavicular ligament will occur at the second step, resulting in an isolated acromioclavicular joint dislocation if clavicle fracture does not occur in the next step; (**D**) on top of a clavicle fracture or acromioclavicular joint dislocation in the second step, a clavicle fracture with associated ipsilateral acromioclavicular joint dislocation will eventually occur if the residual traumatic energy is sufficiently high

Through comprehensive literature review and clinical experience analysis, we propose a three-component injury mechanism hypothesis for MCFs with ipsilateral ACJ injuries: (1) ACJ capsule and ACL disruption, (2) CCL rupture, and (3) clavicle fracture classified as Allman type I [54]. Based on the reviewed literature, there is general consensus that injury to the ACJ capsule and ACL represents the initial event in the injury cascade(Fig. 3A). However, the sequence of subsequent events—whether the clavicle fracture or CCL rupture occurs next—remains controversial. We propose that this sequence is determined by the relative resistance of the clavicle and CCL to traumatic energy, which may vary among individuals. In scenarios where the CCL exhibits greater resistance to trauma than the clavicle, the clavicle fracture occurs first(Fig. 3B). If the residual force is sufficient, subsequent rupture of the CCL may lead to ACJ dislocation(Fig. 3D), otherwise only an isolated clavicle fracture occurred, and the ACJ is likely to show a normal morphology on radiologic findings. Conversely, if the clavicle demonstrates greater resistance, the CCL ruptures first, resulting in ACJ dislocation(Fig. 3C), followed by a clavicle fracture if the remaining force is adequate(Fig. 3D), otherwise the radiologic findings would show an isolated ACJ dislocation without a concurrent clavicle fracture.

This model explains different clinical scenarios, including isolated clavicle fractures with no ACJ dislocation, isolated ACJ dislocation without clavicle fractures, and cases with both injuries. In cases of isolated clavicle fractures, damage to the ACJ capsule and ACL may already be present, with the CCL remaining intact initially but vulnerable to subsequent rupture(Fig. 3B). These cases warrant particular attention, as premature initiation of rehabilitation without addressing the underlying ACJ capsule and ACL injuries may lead to delayed ACJ dislocation. Although not confirmed by cadaveric and biomechanical studies, this hypothesis is supported by two case reports in which Park et al. [29] and Fulton et al. [1] both documented instances of patients developing ACJ dislocations after clavicle fixation.

**Fig. 4:**
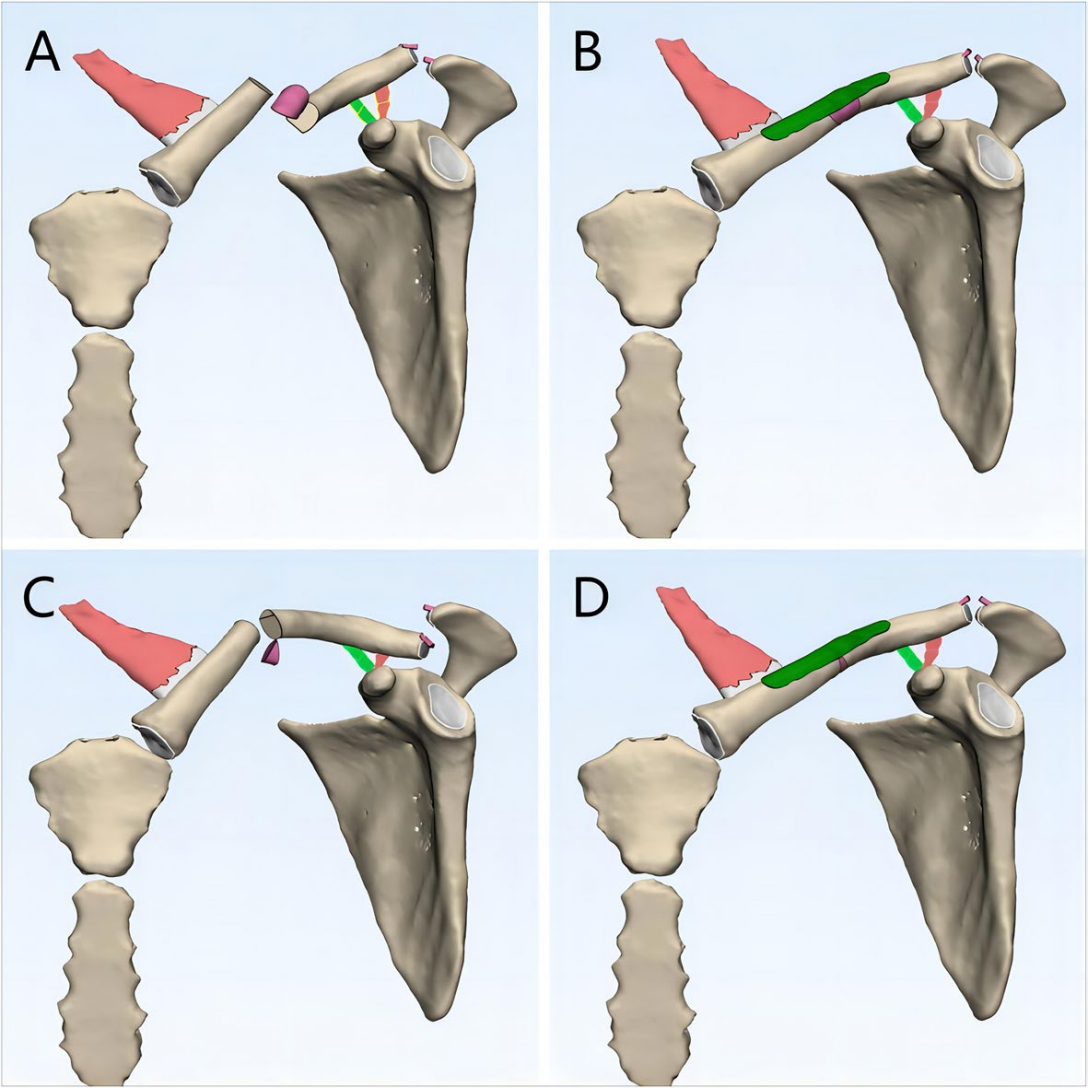
Graphic illustration of the reason why acromioclavicular joint injuries may be overestimated when combined with ipsilateral clavicle fractures. (**A**) With the coracoclavicular ligament intact, the lateral clavicle is displaced superiorly, or it may even be firmly entrapped in the trapezius muscle, demonstrating a pseudo-type III acromioclavicular injury on radiographs and CT; (**B**) however, under these circumstances, the acromioclavicular joint will subsequently get reduced after the completion of the open reduction and internal fixation of clavicle fracture. (**C**) With the coracoclavicular ligament intact, the lateral clavicle is displaced inferiorly, demonstrating a pseudo-type VI acromioclavicular injury on radiographs and CT; (**D**) however, under these circumstances, the acromioclavicular joint will also subsequently get reduced after the completion of the open reduction and internal fixation of clavicle fracture

**Fig. 5:**
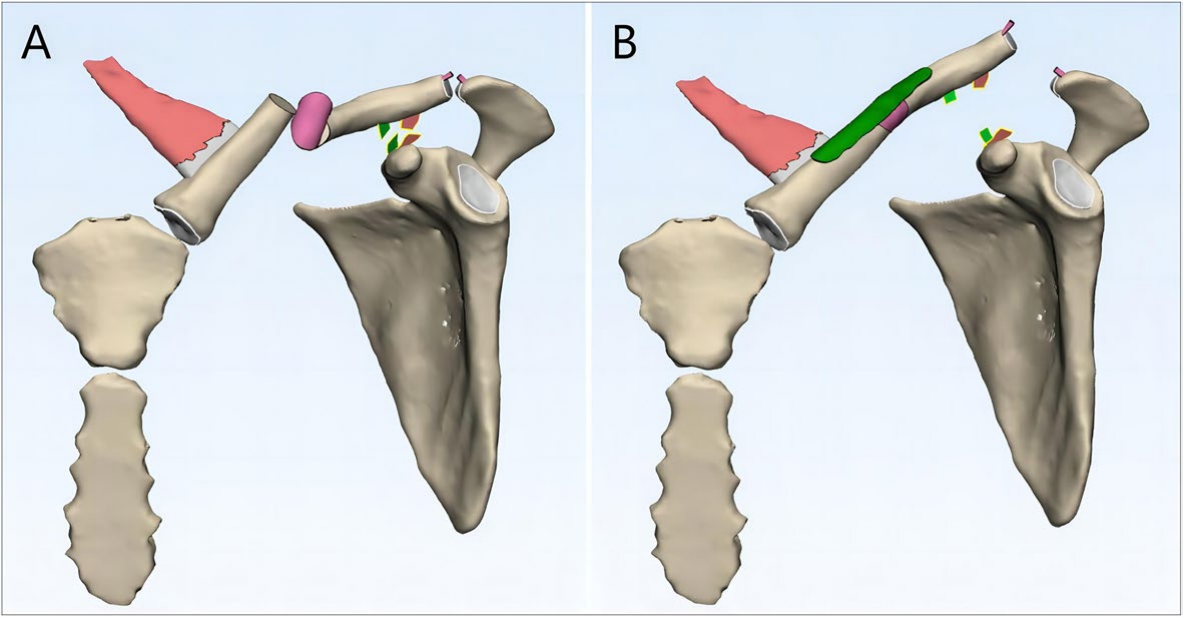
Graphic illustration of the reason why acromioclavicular joint injuries may be underestimated or missed when combined with ipsilateral clavicle fractures. (**A**) When the clavicle fracture and the complete rupture of acromioclavicular ligament and coracoclavicular ligament occur simultaneously, the "floating" lateral clavicle may be displaced downward along with the scapula due to gravity and traction by regional muscles, causing the illusion that acromioclavicular joint is not dislocated on the radiographs and CT; (**B**) under such circumstances, the actual acromioclavicular joint injury becomes apparent after the clavicle has been restored to continuity by internal fixation, resulting in a “delayed” acromioclavicular joint dislocation

The mechanism behind isolated ACJ dislocations is generally understood to involve damage to the ACJ capsule, ACL, and CCL [51, 55–58]. The Rockwood classification [59], which is widely used to classify ACJ injuries, categorizes them into six types based on the involvement of these ligaments and the degree of dislocation. Higher-grade injuries, particularly types III and above, are associated with CCL disruption. However, intraoperative findings in some reviewed cases with type IV and type VI ACJ dislocations revealed intact CCLs [24, 41], challenging the assumption that higher-grade ACJ injuries always correlate with complete CCL rupture. Practically, in cases of MCFs with ipsilateral ACJ injuries, the relationship between ACJ dislocation and CCL injury becomes more complex. For instance, if the CCL remains intact but the lateral clavicle is displaced superiorly, inferiorly, or entrapped in the trapezius muscle, imaging findings may suggest a type III or higher-grade ACJ dislocation. This could lead to an overestimation of the ACJ injury (Fig. 4A, Fig. 4C), as complete CCL rupture would typically be involved in isolated type IV or V dislocations. In such cases, the ACJ is likely to reduce spontaneously following fixation of the clavicle fracture (Fig. 4B, Fig. 4D), as supported by the cases of Davies et al. [31] and Milchteim et al. [44]. Conversely, when the ACJ capsule, ACL, and CCL are all ruptured, the medial clavicle may be displaced superiorly by the pull of the sternocleidomastoid muscle, while the lateral clavicle, due to its "floating" nature, may displace inferiorly along with the scapula under the influence of gravity. This can create the illusion of ACJ integrity on imaging, leading to an underestimation of the injury (Fig. 5A). The true extent of the ACJ dislocation often becomes apparent only after clavicular continuity is restored (Fig. 5B), a phenomenon that may explain the intraoperative "delayed" ACJ dislocation reported by Shih et al. [20].

Notably, it should be emphasized that while our three-component injury mechanism hypothesis for MCF with associated ACJ injuries provides a comprehensive framework for understanding the spectrum of clinical presentations, this model currently lacks robust supporting evidence. Therefore, further well-designed biomechanical studies are warranted to validate this proposed mechanism its clinical implications.

## Clinical examination

The clinical examination for such combined injuries is largely similar to that for isolated MCFs and ACJ dislocations. Visual inspection may reveal superficial abrasions over the posterolateral aspect of the affected shoulder, as well as prominence over the middle third and lateral end of the clavicle. Palpation along the full length of the clavicle typically reveals pain and loss of continuity in the midclavicle, with the most prominent finding being pain upon palpation of the ACJ. Notably, in patients with type IV or VI ACJ dislocations, changes in the appearance of the ACJ may not be easily visible, but bony defects may be palpable [24, 30, 31, 35]. Moreover, the “Piano Key Sign” test is not recommended for diagnosis due to the floating nature of lateral clavicle, which makes the test result typically false negative and unreliable [17, 23]. In contrast, we recommend additional palpation around the coracoid, as tenderness can be commonly found following CCL injuries.

## Imaging

Radiographic evaluation of patients with suspected clavicle fractures should include both anteroposterior and lateral radiographs of the shoulder. Additionally, a radiograph taken with a 45-degree cephalad tilt of the x-ray tube, although not necessary for most patients, can enhance visualization of the clavicle [48, 60]. Since anteroposterior radiographs may not clearly show type IV ACJ injuries, potentially leading to underestimation of distal clavicle displacement, additional axillary lateral radiographs are recommended to assess whether the distal clavicle is displaced posteriorly [24, 30]. Furthermore, as suggested by Rockwood et al. [48], radiographs of the ACJ should be performed with about one-third of the kilovoltage typically used for glenohumeral joint radiographs.

Computer tomography (CT) of the shoulder, including 3D reconstructions, can provide a clear view of the direction and extent of MCF displacement and ACJ dislocations, offering a comprehensive assessment of the pathoanatomy. CT can also identify injuries that are not visible on radiographs, such as type IV ACJ injuries and linear clavicle fractures, which are often underestimated on anteroposterior radiographs. Additionally, CT can detect other associated injuries, including acromion fractures, coracoid fractures, and SCJ injuries. Based on the literature reviewed, we found that concurrent rib fractures and hemopneumothorax were quite common in such combined injuries [14, 17, 21, 31, 38, 40]. Therefore, we recommend extending the CT scan to cover the entire chest, including the contralateral shoulder, to ensure a thorough evaluation.

## Classification

There is limited knowledge regarding the classification of MCFs combined with ipsilateral ACJ injuries. Although Bakir et al.'s [26] classification system for bipolar clavicle injuries includes such combined injuries as type Ib, it only reflects the injury's location and does not address the severity of each individual injury. Clinically, the two injuries are currently classified separately, as if they occurred in isolation. In the Allman classification system [54], clavicle fractures are categorized based on the location of the fracture, with the clavicle divided into three equal parts, and fractures in the middle third classified as Allman type I. Some researchers have further sub-classified medial-third and lateral-third clavicle fractures, but no additional classification or subtypes for MCFs have been reported [6, 61].

On the other hand, the most commonly used classification for ACJ injuries is the Rockwood system [3, 51, 55–59], which categorizes injuries into six types based on ligament involvement, as well as the degree and direction of distal clavicle displacement, with higher grades indicating more severe injuries. Additionally, some researchers [24, 31, 34] have further categorized type VI ACJ dislocations into type VIa (subacromial, supracoracoid) and VIb (subcoracoid) based on the position of the lateral clavicle relative to the coracoid.

Currently, ACJ injuries in combined cases are still reported using the Rockwood classification. However, as discussed in our “Pathoanatomy and mechanism of injury” section, preoperative ACJ classification does not always accurately reflect the true extent of injury. Therefore, a new classification system specifically addressing the severity of MCFs with ipsilateral ACJ injuries is urgently needed to guide treatment decisions.Currently, ACJ injuries in combined cases are generally classified according to the Rockwood classification system. However, as discussed in the Pathoanatomy section, preoperative ACJ classification sometimes fails to accurately capture the full extent of the injury. Consequently, there is an urgent need for a new classification system that can specifically address the severity of both MCF and concomitant ipsilateral ACJ injury, to more effectively guide treatment decisions.

## Treatment

Both isolated clavicle fractures and ACJ dislocations can be treated with either conservative or surgical treatment, depending on the severity of the injury. However, due to their rarity, there are limited cohort studies on cases involving both injuries simultaneously, and no gold standard treatment has been established. In clinical practice, these injuries are still typically managed as two separate isolated injuries. The treatment modalities in the reviewed studies are summarized in Table 2.

Among 39 patients reported in 34 case studies, 27 underwent bilateral surgical treatment, 4 received bilateral conservative management, 5 were treated conservatively for the clavicle fracture while undergoing surgery for the ACJ dislocation, and 3 received surgery for the clavicle fracture while being treated conservatively for the ACJ dislocation. Regarding the choice of fixation implants, plates and screws remain the primary approach for treating clavicle fractures. Of the 30 patients who underwent surgery for clavicle fractures, 26 received plate fixation, 2 were treated with intramedullary fixation using Knowles pins [20, 44], and the fixation implants were unspecified for 2 patients [46, 48]. The predominance of plate fixation can be attributed to the difficulty of achieving closed reduction in clavicle fractures due to the floating nature of the lateral clavicle in such combined injuries, which makes intramedullary fixation less feasible. Furthermore, intramedullary implants occupy the intramedullary space of the clavicle, limiting the implantation of hardware for coracoclavicular fixation and further restricting its use. To address this challenge, future development of intramedullary fixation systems specifically designed for combined injuries may prove beneficial. These systems could function similarly to intramedullary nail systems used for long bone fractures, enabling precise coracoclavicular fixation through an external guide following clavicle fracture fixation.

In contrast, among 32 patients who underwent surgery for ACJ dislocation, 14 received coracoclavicular fixation only, 12 received isolated acromioclavicular fixation only, and 5 underwent both coracoclavicular and acromioclavicular fixation. Based on the type of internal fixation used, the fixation methods can be classified as rigid fixation and elastic fixation. A common form of rigid fixation is the hook plate for distal clavicle. However, this approach has notable drawbacks, including the proven risk of subacromial osteolysis [62, 63]. Additionally, in the treatment of combined injuries, a positional conflict may arise between the hook plate used for ACJ fixation and the plate used for clavicle fracture stabilization. This positional conflict could theoretically lead to uneven stress distribution, resulting in stress fractures of the clavicle. However, such a complication has not been observed in the limited number of cases reviewed. To prevent stress fractures at the junction of the two plates, Wijdicks et al. [40] placed a reconstruction plate on the anterior clavicle surface, centered at the junction, which successfully promoted bony healing of the clavicle fracture and led to a satisfactory clinical outcome.

In recent years, with advancements in arthroscopic techniques and biomechanical research, elastic coracoclavicular fixation has gradually become the preferred method for managing ACJ dislocations. Representative fixation methods include the Endobutton, dog bone button, TightRope system, suture anchors, and autologous or allogeneic tendon grafts. These newer technologies and implants offer significant advantages, including a more minimally invasive surgical approach and the elimination of the need for secondary removal of metal implants. These innovations will provide valuable insights and guidance for future research, ultimately advancing the development of improved management strategies for combined injuries.

## Complications and prognosis

Complications and prognosis are related to a variety of factors, including injury mechanism, injury severity, treatment modalities, surgical procedures, and postoperative rehabilitation. For isolated clavicle fractures, common complications include nonunion, malunion with an abnormal appearance, superficial or deep infection, numbness following iatrogenic supraclavicular nerve injuries, hardware failures, and re-fracture after hardware removal [8, 60]. Similarly, for isolated ACJ injuries, common complications include hardware failures, neurovascular injuries, ACJ degeneration, continued pain, and iatrogenic coracoid fractures [1]. Interestingly, however, in the case reports we reviewed, only a small number of patients who suffered such combined injuries experienced one or more of these complications, possibly due to the small patient base. Detailed information is provided in Table 2.

Among the 39 patients reviewed, 37 had documented complications, with 11 patients experiencing complications [13, 16, 21, 25, 29, 34, 36, 38–40, 42]. Five of these complications occurred following conservative treatment for clavicle fracture, ACJ dislocation, or both [16, 21, 29, 34, 39], while six arose after surgical treatment for both injuries [13, 25, 36, 38, 40, 42]. The complications were primarily concentrated in the ACJ, including ACJ osteoarthritis and the persistent widening. Notably, among all patients who received conservative treatment for ACJ injuries, only one did not experience any complications [31], suggesting that conservative management of ACJ dislocations is more prone to complications. However, among the patients who experienced ACJ-related complications, only one underwent further surgical intervention, which involved distal clavicle excision.

Regardless of the treatment approach, all clavicle fractures ultimately achieved bony union. The only complication reported in relation to clavicle fractures was malunion, resulting in a deformity, which occurred in three patients who received conservative treatment [21, 34, 39]. No complications were reported in patients who underwent clavicle fracture fixation. However, due to the lack of detailed information on healing times in the reviewed studies, it is not possible to make a definitive comparison of the effectiveness of conservative versus surgical treatment for clavicle fractures in the context of combined injuries. Therefore, future research should not only focus on fracture healing and complication rates, but also consider factors such as treatment duration, healing times, and patients' subjective experiences during treatment, in order to provide a more comprehensive and objective comparison.

Remarkably, all the reviewed patients ultimately achieved satisfactory outcomes, regardless of whether they were treated conservatively or surgically. Even those who developed complications were able to attain a fairly satisfactory range of motion during short- and medium-term follow-up [16, 29, 34, 38, 42]. However, due to the limited number of studies that used standardized scoring systems to quantify patient outcomes, and the lack of consistency in the scoring tools employed, it is challenging to provide a comprehensive quantitative analysis of the overall prognosis for this combined injury. This highlights the need for future studies to employ objective methods for assessing prognosis in order to enhance the value and reliability of research findings. Additionally, the detailed recording of limb range of motion, including the extent of abduction, adduction, flexion, extension, and rotation, should also be a focus of future studies.

## Limitations

There are several limitations in our study. First, with regard to the search methodology, we limited our search to three commonly used medical literature databases, which inevitably led to the omission of some relevant studies, such as conference abstracts, unpublished manuscripts, or papers that have been published but are not yet indexed in the databases. Furthermore, due to constraints related to language, network access, and policy, we were unable to obtain articles from non-international journals published in different countries. Second, as our study focuses on a rare type of injury, we could only extract relevant data from a very limited number of case reports with short follow-up periods, which results in a lower level of evidence for our findings. Additionally, due to the variation in treatment approaches and outcome measures, we were unable to pool the data for a meta-analysis to derive more objective conclusions. Nevertheless, to the best of our knowledge, this is the most comprehensive summary of this rare injury to date, which may contribute to increased awareness and ultimately benefit patients. Third, in our discussion of the injury mechanism, we proposed a hypothesis based on the existing literature and our clinical experience. However, this hypothesis is not currently supported by any direct evidence, and further validation through biomechanical research, cadaveric studies, or finite element analysis is needed in the future.

## Conclusion

MCFs with ipsilateral ACJ injuries are rare and often missed when the ACJ injuries are mild. This can lead to progressive dislocation, arthritis, and pain, often necessitating revision treatments. These combined injuries typically result from high-energy trauma and are frequently associated with comorbidities such as rib fractures, hemopneumothorax, scapula fractures, and neurovascular injuries, along with atypical MCF displacement patterns. Therefore, MCF with such comorbidities or atypical displacements should raise suspicion of combined injuries. Notably, due to the "floating" nature of the lateral clavicle, the "Piano Key Sign" is often negative and should not be used for diagnosis. Initial ACJ evaluation can also be unreliable, so reevaluation after MCF fixation is strongly recommended to determine the final treatment plan. Type IV ACJ injuries can be underestimated on anteroposterior radiographs, and additional axillary radiographs and CT scans are recommended to better visualize posterior clavicle displacement.

Regarding the injury sequence, it is widely believed that damage to the ACJ capsule and ligaments occurs first, but is insufficient to cause significant dislocation. We hypothesize that the sequence of CCL injury and MCF depends on their respective trauma tolerance. This suggests that an isolated MCF may actually involve concurrent ACJ capsule and ligament injuries, with the CCL remaining intact. However, this hypothesis requires validation through future studies.

Based on a limited number of cases with follow-up of no more than two years, serious complications are rare in such combined injuries, regardless of whether treated conservatively or surgically. The majority of patients had favorable outcomes, and even those who experienced complications generally achieved a satisfactory range of motion. To better understand the long-term prognosis of such combined injuries, future studies should involve larger patient cohorts with follow-up of at least three years.

## Data Availability

No datasets were generated or analysed during the current study.

## Abbreviations

MCF: Midshaft clavicle fractures
ACJ: Acromioclavicular joint
SCJ: Sternoclavicular joint
ACL: Acromioclavicular ligament
CCL: Coracoclavicular ligament
JBI: Joanna Briggs Institute
CT: Computer tomography

## References

[1] Fulton ZW, Singleton A, Miller RM. Coracoclavicular Ligament Reconstruction Using TightRope for Delayed Grade III Acromioclavicular Joint Injury After Ipsilateral Diaphyseal Clavicle Fracture Fixation: Surgical Technique and Review of Current Literature. Tech Hand Up Extrem Surg. 2022;26(3):208–11. 10.1097/BTH.0000000000000386. 35698303 10.1097/BTH.0000000000000386

[2] Mohammed KD, Stachiw D, Malone AA. Type IV acromioclavicular joint dislocation associated with a mid-shaft clavicle malunion. Int J Shoulder Surg. 2016;10(1):37–40. 10.4103/0973-6042.174518. 26980988 10.4103/0973-6042.174518 PMC4772415

[3] Lindborg CM, Smith RD, Reihl AM, Bacevich BM, Cote M, O’Donnell E, Mazzocca AD, Hutchinson I. Current Concepts in Management of Acromioclavicular Joint Injury. J Clin Med. 2024;13(5):1413. 10.3390/jcm13051413. 38592250 10.3390/jcm13051413 PMC10931774

[4] Muench LN, Berthold DP, Rupp MC, Dorsey CG, Hawthorne B, Trudeau MT, Wolf JD, Wellington I, Mazzocca AD. Long-Term Functional Outcomes and Athletic Ability in Shoulder Sports After Anatomic Coracoclavicular Ligament Reconstruction for Chronic Type 3 and 5 Acromioclavicular Joint Injuries. Orthop J Sports Med. 2024;12(2):23259671241227224. 10.1177/23259671241227224. 38313753 10.1177/23259671241227224 PMC10836141

[5] Verstraete O, Van Tongel A, De Wilde L, Peeters I. Acromioclavicular reconstruction techniques after acromioclavicular joint injuries: A systematic review of biomechanical studies. Clin Biomech (Bristol, Avon). 2023;101: 105847. 10.1016/j.clinbiomech.2022.105847. 10.1016/j.clinbiomech.2022.105847 36521410

[6] von Rüden C, Rehme-Röhrl J, Augat P, Friederichs J, Hackl S, Stuby F, Trapp O. Evidence on treatment of clavicle fractures. Injury. 2023;54(Suppl 5): 110818. 10.1016/j.injury.2023.05.049. 37217399 10.1016/j.injury.2023.05.049

[7] Serpico M, Tomberg S. The emergency medicine management of clavicle fractures. Am J Emerg Med. 2021;49:315–25. 10.1016/j.ajem.2021.06.011. 34217972 10.1016/j.ajem.2021.06.011

[8] Markes AR, Garcia-Lopez E, Halvorson RT, Swarup I. Management of Displaced Midshaft Clavicle Fractures in Pediatrics and Adolescents: Operative vs Nonoperative Treatment. Orthop Res Rev. 2022;14:373–81. 10.2147/ORR.S340538. 36345395 10.2147/ORR.S340538 PMC9636878

[9] Saragaglia D, Refaie R. Displaced mid-shaft clavicular fractures: state of the art for athletes and young active people. Int Orthop. 2021;45(10):2679–86. 10.1007/s00264-021-05113-2. 34309695 10.1007/s00264-021-05113-2

[10] Page MJ, McKenzie JE, Bossuyt PM, et al. The PRISMA 2020 statement: an updated guideline for reporting systematic reviews. Syst Rev. 2021;10(1):89. 10.1186/s13643-021-01626-4. 33781348 10.1186/s13643-021-01626-4 PMC8008539

[11] Parmar MK, Torri V, Stewart L. Extracting summary statistics to perform meta-analyses of the published literature for survival endpoints. Stat Med. 1998;17(24):2815–34. 10.1002/(sici)1097-0258(19981230)17:24%3c2815::aid-sim110%3e3.0.co;2-8. 9921604 10.1002/(sici)1097-0258(19981230)17:24<2815::aid-sim110>3.0.co;2-8

[12] Moola S, Munn Z, Tufanaru C, Aromataris E, Sears K, Sfetcu R, Currie M, Qureshi R, Mattis P, Lisy K, Mu P-F. Chapter 7: Systematic reviews of etiology and risk. In: Aromataris E, Munn Z (Editors). JBI Manual for Evidence Synthesis. JBI, 2020. Available from https://synthesismanual.jbi.global.

[13] López Palacios C, Sanchez-Munoz E, Pipa Muñiz I, Rodríguez García N, Maestro Fernández A. Simultaneous Clavicle Fracture and Acromioclavicular Joint Dislocation: Novel Surgical Technique: A Case Report. JBJS Case Connect 2021;11(2):e20.00775. 10.2106/JBJS.CC.20.00775. 10.2106/JBJS.CC.20.00775 34161305

[14] Prasetia R, Rasyid HN. Bipolar fracture dislocation of clavicle: A report of osteosynthesis and early soft tissue reconstruction. Int J Surg Case Rep. 2017;41:194–9. 10.1016/j.ijscr.2017.10.025. 29096342 10.1016/j.ijscr.2017.10.025 PMC5683890

[15] Ottomeyer C, Taylor BC, Isaacson M, Martinez L, Ebaugh P, French BG. Midshaft clavicle fractures with associated ipsilateral acromioclavicular joint dislocations: Incidence and risk factors. Injury. 2017;48(2):469–73. 10.1016/j.injury.2016.12.021. 28062098 10.1016/j.injury.2016.12.021

[16] Kembhavi RS, James B. Ipsilateral Closed Clavicle and Scapular spine Fracture with Acromioclavicular Joint Disruption. J Orthop Case Rep 2015;5(2):58–61. 10.13107/jocr.2250-0685.276. 10.13107/jocr.2250-0685.276 PMC4722593 27299047

[17] Gao Z, Cai P, Yao K, Long N, Liu L, Xiao C. Mid-clavicle fracture with dislocation of the ipsilateral acromioclavicular joint treated with Endobutton system: A case report and review of the literature. Medicine (Baltimore). 2021;100(47): e27894. 10.1097/MD.0000000000027894. 34964758 10.1097/MD.0000000000027894 PMC8615337

[18] Cosic F, Ernstbrunner L, Hoy GA, Ooi KS, Ek ET. Case Report: Midshaft clavicle fracture with concomitant high grade (Type V) acromioclavicular joint dislocation. Front Surg. 2022;9: 885378. 10.3389/fsurg.2022.885378. 36017522 10.3389/fsurg.2022.885378 PMC9395734

[19] Marjoram TP, Chakrabarti A. Segmental clavicle fracture and acromio-clavicular joint disruption: an unusual case report. Shoulder Elbow. 2015;7(3):187–9. 10.1177/1758573214564496. 27582977 10.1177/1758573214564496 PMC4935151

[20] Shih YJ, Chang HC, Wu CL. Optimizing Treatment for Combined Midshaft Clavicle Fracture and Acromioclavicular Joint Injury: A Case Study Highlighting the Efficacy of Knowles Pin Fixation. Am J Case Rep 2023;24:e939325. 10.12659/AJCR.939325. 10.12659/AJCR.939325 PMC10254087 37277979

[21] Lee KW, Bae JY, Seo DK, Ha JK, Ra HJ, Kim JH, Ho BC. Bipolar Injury of the Clavicle. Orthopedics. 2018;41(5):e681–8. 10.3928/01477447-20180724-02. 30052261 10.3928/01477447-20180724-02

[22] Solooki S, Azad A. Simultaneous middle third clavicle fracture and type 3 acromioclavicular joint dislocation; a case report. Arch Bone Jt Surg. 2014Mar;2(1):69–71. 25207318 PMC4151440

[23] van de Voort JC, van Doesburg PG, Leijnen M. Ipsilateral Rockwood type IV acromioclavicular joint dislocation and midshaft clavicle fracture: a case report and review of the literature. JSES Rev Rep Tech. 2022;3(2):236–41. 10.1016/j.xrrt.2022.11.007. 37588430 10.1016/j.xrrt.2022.11.007 PMC10426522

[24] Grossi EA, Macedo RA. Acromioclavicular dislocation type VI associated with diaphyseal fracture of the clavicle. Rev Bras Ortop. 2013;48(1):108–10. 10.1016/j.rboe.2011.12.002. 31304120 10.1016/j.rboe.2011.12.002 PMC6565910

[25] Schots JP, van Laarhoven SN, Hustinx PA, Pijnenburg AM, Meesters B, de Loos ER. Surgical treatment of acromioclavicular dislocation associated with midshaft fracture of the ipsilateral clavicle. Acta Orthop Belg. 2020;86(3):532–8. 33581039

[26] Bakir MS, Carbon R, Ekkernkamp A, Schulz-Drost S. Monopolar and Bipolar Combination Injuries of the Clavicle: Retrospective Incidence Analysis and Proposal of a New Classification System. J Clin Med. 2021;10(24):5764. 10.3390/jcm10245764. 34945058 10.3390/jcm10245764 PMC8706334

[27] Sharma N, Mandloi A, Agrawal A, Singh S. Acromioclavicular Joint Dislocation with Ipsilateral Mid Third Clavicle, Mid Shaft Humerus and Coracoid Process Fracture - A Case Report. J Orthop Case Rep 2016;6(2):24–27. 10.13107/jocr.2250-0685.414. 10.13107/jocr.2250-0685.414 PMC5040563 27703932

[28] Psarakis SA, Savvidou OD, Voyaki SM, Beltsios M, Kouvaras JN. A rare injury of ipsilateral mid-third clavicle fracture with acromioclavicular joint dislocation. Hand (N Y). 2011;6(2):228–32. 10.1007/s11552-011-9323-y. 22654711 10.1007/s11552-011-9323-y PMC3092883

[29] Park CH, Shon OJ, Seo JS, Kim GB. Midshaft clavicle fracture with ipsilateral acromioclavicular joint separation found during serial follow-up. J Orthop Sci. 2016;21(3):399–402. 10.1016/j.jos.2015.06.007. 26740446 10.1016/j.jos.2015.06.007

[30] Yeh PC, Miller SR, Cunningham JG, Sethi PM. Midshaft clavicle fracture and acromioclavicular dislocation: a case report of a rare injury. J Shoulder Elbow Surg. 2009;18(5):e1–4. 10.1016/j.jse.2008.09.011. 19046642 10.1016/j.jse.2008.09.011

[31] Davies EJ, Fagg JA, Stanley D. Subacromial, supracoracoid dislocation of the acromioclavicular joint with ipsilateral clavicle fracture: a case report with review of the literature and classification. JRSM Open. 2014;5(7):2054270414527281. 10.1177/2054270414527281. 25057405 10.1177/2054270414527281 PMC4100230

[32] Chillemi C, Franceschini V, Dei Giudici L, Alibardi A, Salate Santone F, Ramos Alday LJ, Osimani M. Epidemiology of isolated acromioclavicular joint dislocation. Emerg Med Int. 2013;2013: 171609. 10.1155/2013/171609. 23431452 10.1155/2013/171609 PMC3568861

[33] Madi S, Pandey V, Khanna V, Acharya K. A dual injury of the shoulder: acromioclavicular joint dislocation (type IV) coupled with ipsilateral mid-shaft clavicle fracture. BMJ Case Rep 2015;2015:bcr2015213254. 10.1136/bcr-2015-213254. 10.1136/bcr-2015-213254 PMC4680282 26598529

[34] Juhn MS, Simonian PT. Type VI acromioclavicular separation with middle-third clavicle fracture in an ice hockey player. Clin J Sport Med. 2002;12(5):315–7. 10.1097/00042752-200209000-00011. 12394206 10.1097/00042752-200209000-00011

[35] Dong D, Yu M, Gu G. Simultaneous bilateral midshaft clavicle fractures with unilateral dislocation of the acromioclavicular joint: A case report. Medicine (Baltimore). 2017;96(21): e6975. 10.1097/MD.0000000000006975. 28538398 10.1097/MD.0000000000006975 PMC5457878

[36] Sebesta P, Hach J, Tlustý SZ. Middle-third clavicle fracture with ipsilateral acromioclavicular dislocation [in Czech]. Acta Chir Orthop Traumatol Cech. 2014;81(3):238–40. 24945394

[37] Itami Y, Shirahata T. Surgical treatment of clavicle fracture and dislocation of acromioclavicular joint [in Japanese]. Shujutsu. 1971;25(6):750–64. 5567671

[38] Beytemür O, Adanir O, Dinçel YM, Baran MA, Güleç MA. Clavicle diaphyseal fracture, ipsilateral type 3 acromioclavicular joint dislocation stabilized with double plate. Int J Shoulder Surg. 2013;7(4):153–4. 10.4103/0973-6042.123536. 24403764 10.4103/0973-6042.123536 PMC3883191

[39] Heinz WM, Misamore GW. Mid-shaft fracture of the clavicle with grade III acromioclavicular separation. J Shoulder Elbow Surg. 1995;4(2):141–2. 10.1016/s1058-2746(05)80069-2. 7600166 10.1016/s1058-2746(05)80069-2

[40] Wijdicks CA, Anavian J, Ly TV, Spiridonov SI, Craig MR, Cole PA. Surgical management of a midshaft clavicle fracture with ipsilateral acromioclavicular dislocation: A report on 2 cases and review of the literature. Injury Extra. 2013;44(2):9–12. 10.1016/j.injury.2012.09.007.

[41] Wisniewski TF. Posterior Acromioclavicular Dislocation with Clavicular Fracture and Trapezius Entrapment. Eur J Trauma. 2004;30(2):120–3. 10.1007/s00068-004-1353-5.

[42] Sobhani Eraghi A, Moghtadaei M, Azizpour I, Hajializade M. Simultaneous Injury of Mid-shaft Clavicle Fracture and Type IV Acromioclavicular Joint Dislocation. JROS 2020; 7(1):35–40. 10.32598/JROSJ.7.1.35.

[43] Lancourt JE. Acromioclavicular dislocation with adjacent clavicular fracture in a horseback rider. A case report. Am J Sports Med 1990;18(3):321–322. 10.1177/036354659001800317. 10.1177/036354659001800317 2372085

[44] Milchteim C, Doppelt JD, Neviaser RJ. Subacromial dislocation of the acromioclaviclular joint with associated fracture of the clavicle. J Shoulder Elbow Surg. 2018;27(10):e297–9. 10.1016/j.jse.2018.05.045. 30076037 10.1016/j.jse.2018.05.045

[45] Kakwani RG, Tourret LJ. Fracture Clavicle with Acromioclavicular Dislocation: A Complex Injury. Should Elb. 2011;3(1):31–3. 10.1111/j.1758-5740.2010.00102.x.

[46] Woolf SK, Valentine BJ, Barfield WR, Hartsock LA. Middle-third clavicle fracture with associated type IV acromioclavicular separation: case report and literature review. J Surg Orthop Adv. 2013;22(2):183–6. 10.3113/jsoa.2013.0183. 23628577 10.3113/jsoa.2013.0183

[47] Tidwell JE, Kennedy PM, McDonough EB. Concurrent treatment of a middle-third clavicle fracture and type IV acromioclavicular dislocation. Am J Orthop (Belle Mead NJ). 2014;43(11):E275–8. 25379757

[48] Wurtz LD, Lyons FA, Rockwood CA Jr. Fracture of the middle third of the clavicle and dislocation of the acromioclavicular joint. A report of four cases. J Bone Joint Surg Am 1992;74(1):133–137. 1734003

[49] Subramanyam KN, Mundargi AV, Gopakumar KU, Bharath T, Prabhu MV, Khanchandani P. Displaced midshaft clavicle fractures in adults - is non-operative management enough? Injury. 2021;52(3):493–500. 10.1016/j.injury.2020.10.019. 33066986 10.1016/j.injury.2020.10.019

[50] Patted SM, Kumar A, Halawar RS. Morphometric Analysis of Clavicle and Its Medullary Canal: A Computed Tomographic Study. Indian J Orthop. 2020;54(Suppl 2):283–91. 10.1007/s43465-020-00223-2. 33194103 10.1007/s43465-020-00223-2 PMC7609512

[51] Mansour J, Nassar JE, Estephan M, Boulos K, Daher M. Acromioclavicular joint dislocation and concomitant labral lesions: a systematic review. Clin Shoulder Elb. 2024;27(2):247–53. 10.5397/cise.2023.00640. 38303595 10.5397/cise.2023.00640 PMC11181061

[52] Shah SS, Ferkel E, Mithoefer K. High Prevalence of Superior Labral Anterior-Posterior Tears Associated With Acute Acromioclavicular Joint Separation of All Injury Grades. Orthop J Sports Med. 2020;8(8):2325967120941850. 10.1177/2325967120941850. 32923497 10.1177/2325967120941850 PMC7457667

[53] Okano I, Sawada T, Inagaki K. Bipolar Dislocation of the Clavicle: A Report of Two Cases with Different Injury Patterns and a Literature Review. Case Rep Orthop. 2017;2017:2935308. 10.1155/2017/2935308. 29527368 10.1155/2017/2935308 PMC5763060

[54] Allman FL Jr. Fractures and ligamentous injuries of the clavicle and its articulation. J Bone Joint Surg Am. 1967;49(4):774–84. 6026010

[55] Nolte PC, Lacheta L, Dekker TJ, Elrick BP, Millett PJ. Optimal Management of Acromioclavicular Dislocation: Current Perspectives. Orthop Res Rev. 2020;12:27–44. 10.2147/ORR.S218991. 32184680 10.2147/ORR.S218991 PMC7062404

[56] Bieling M, Ellwein A, Lill H, Sehmisch S, Reeh FM. Proximal humerus fracture and acromioclavicular joint dislocation. Innov Surg Sci. 2024;9(2):67–82. 10.1515/iss-2023-0049. 39100718 10.1515/iss-2023-0049 PMC11294519

[57] Roberts MA, Wall C. Acromioclavicular dislocation: A common injury affecting a healthy population. Aust J Gen Pract 2024;53(8):571–573. 10.31128/AJGP-07-23-6909. 10.31128/AJGP-07-23-6909 39099125

[58] Navarro R, Kody M, Chapek M, Combs K. Acromioclavicular joint dislocation: a novel surgical technique for acromioclavicular joint reduction with coracoclavicular ligament reconstruction and anatomic conoid ligament reconstruction. JSES Rev Rep Tech. 2024;4(2):213–21. 10.1016/j.xrrt.2023.12.001. 38706662 10.1016/j.xrrt.2023.12.001 PMC11065762

[59] Edgar C, DeGiacomo A, Lemos MJ, Mazzocca AD. Acromioclavicular joint injuries. In: Court-Brown CM, Heckman JD, McQueen MM, Ricci WM, Tornetta IIIP, editors. ROCKWOOD AND GREEN’S Fractures in Adults, vol. 1. Philadelphia: Wolters Kluwer Health; 2015. p. 1573–606.

[60] Wiesel B, Nagda S, Mehta S, Churchill R. Management of Midshaft Clavicle Fractures in Adults. J Am Acad Orthop Surg. 2018;26(22):e468–76. 10.5435/JAAOS-D-17-00442. 30180095 10.5435/JAAOS-D-17-00442

[61] Kim DW, Kim DH, Kim BS, Cho CH. Current Concepts for Classification and Treatment of Distal Clavicle Fractures. Clin Orthop Surg. 2020;12(2):135–44. 10.4055/cios20010. 32489533 10.4055/cios20010 PMC7237254

[62] Chen YT, Wu KT, Jhan SW, et al. Is coracoclavicular reconstruction necessary in hook plate fixation for acute unstable acromioclavicular dislocation? BMC Musculoskelet Disord. 2021;22(1):127. 10.1186/s12891-021-03978-3. 33522921 10.1186/s12891-021-03978-3 PMC7849128

[63] Lee CY, Chen PC, Liu YC, et al. Does coracoclavicular augmentation additional to hook plate fixation provide benefits in acute unstable acromioclavicular dislocation? A meta-analysis. BMC Musculoskelet Disord. 2022;23(1):205. 10.1186/s12891-022-05142-x. 35246100 10.1186/s12891-022-05142-x PMC8897880

